# Inflammatory cytokines mediating the effect of oral lichen planus on oral cavity cancer risk: a univariable and multivariable mendelian randomization study

**DOI:** 10.1186/s12903-024-04104-0

**Published:** 2024-03-22

**Authors:** Tao Zheng, Chengyong Liu, Yetong Wang, Han Zhou, Rong Zhou, Xuan Zhu, Zibing Zhu, Yisi Tan, Zhengrui Li, Xufeng Huang, Jin Tan, Keke Zhu

**Affiliations:** 1 Department of Stomatology, The First Affiliated Hospital of Hunan University of Chinese Medicine, Changsha, Hunan China; 2 Hunan University of Traditional Chinese Medicine, Changsha, Hunan China; 3 Changsha Hospital for Maternal and Child Health Care, Changsha, Hunan China; 4 College of Stomatology, Shanghai Jiao Tong University, Shanghai, China; 5 Faculty of Dentistry, University of Debrecen, Debrecen, Hungary

**Keywords:** Oral lichen planus, Oral cavity cancer, Mendelian randomisation, Inflammatory cytokines, Causal inference

## Abstract

**Background:**

While observational studies and experimental data suggest a link between oral lichen planus (OLP) and oral cavity cancer (OCC), the causal relationship and the role of inflammatory cytokines remain unclear.

**Methods:**

This study employed a univariable and multivariable Mendelian Randomization (MR) analysis to investigate the causal relationship between OLP and the risk of OCC. Additionally, the potential role of inflammatory cytokines in modulating this association was explored. Instrumental variables were derived from genetic variants associated with OLP (*n* = 377,277) identified in Finngen R9 datasets, with 41 inflammatory cytokines as potential mediators, and OCC (*n* = 4,151) as the outcome variable. Analytical methods including Inverse Variance Weighted (IVW), Weighted Median, MR-Egger, and MR-PRESSO were utilized to assess the causal links among OLP, inflammatory cytokines, and OCC risk. Multivariable MR (MVMR) was then applied to quantify the mediating effects of these cytokines in the relationship between OLP and increased OCC risk.

**Results:**

MR analysis provided strong evidence of a causal relationship between OLP (OR = 1.417, 95% CI = 1.167–1.721, *p* < 0.001) and the risk of OCC. Furthermore, two inflammatory cytokines significantly influenced by OLP, IL-13 (OR = 1.088, 95% CI: 1.007–1.175, *P* = 0.032) and IL-9 (OR = 1.085, 95% CI: 1.005–1.171, *P* = 0.037), were identified. Subsequent analysis revealed a significant causal association only between IL-13 (OR = 1.408, 95% CI: 1.147–1.727, *P* = 0.001) and higher OCC risk, establishing it as a potential mediator. Further, MVMR analysis indicated that IL-13 (OR = 1.437, 95% CI = 1.139–1.815, *P* = 0.002) mediated the relationship between OLP and OCC, accounting for 8.13% of the mediation.

**Conclusion:**

This study not only elucidates the potential causal relationship between OLP and the risk of OCC but also highlights the pivotal mediating role of IL-13 in this association.

**Supplementary Information:**

The online version contains supplementary material available at 10.1186/s12903-024-04104-0.

## Introduction

Head and neck squamous cell carcinoma (HNSCC), is recognized worldwide as the sixth most prevalent malignancy, has its larger subgroup in oral cavity cancer (OCC) [1, 2]. Despite the progress in treating OCC in recent years, the overall 5-year survival rate for OCC patients continues to be about 50% [3], underscoring the urgent need for effective prevention and early detection strategies [4]. The World Health Organization (WHO) has defined oral potentially malignant disorders (OPMD) as “a heterogeneous group of diseases clinically characterized, associated with a variable risk of progression to oral squamous cell carcinoma (OSCC)“ [5]. This highlights the critical role of OPMDs, such as oral lichen planus (OLP) or oral leukoplakia (OL), in the continuum of OCC prevention and underscores the necessity of understanding the transition from OPMD to OCC.

OLP is a common T-cell-mediated chronic autoimmune inflammatory [6]. Over the past decade, the incidence and prevalence of OLP have consistently increased, making it a global public health concern [7]. Moreover, Such chronic inflammatory disease is intimately associated with the onset of tumors and the progression of cancer [8]. Given this context, the elevated risk of OCC in patients with OLP warrants careful attention, classifying OLP as an OPMD with a higher malignancy rate [9]. Despite various epidemiological studies assessing the relationship between OLP and OCC, inconsistencies in findings highlight the complexity of this transition and the need for more rigorous investigation [10, 11]. A meta-analysis incorporating data of 20,092 OLP patients indicates that only a minority of OLP patients progress to Oral squamous cell carcinoma (OSCC) [12]. However, another updated meta-analysis, which included data from 26,742 OLP patients, supports the idea that the malignancy transformation rate of OLP has been underestimated [13]. Moreover, the progression from OLP to OCC involves a complex inflammatory process that has not yet been clarified [14]. Recognizing the role of inflammation in this transition is also crucial, as it may identify potential targets for intervention and prevention.

Previous studies have supported the role of inflammatory mediators in the pathogenesis of OLP to OCC [15]. Among these mediators, interleukins have been identified as key contributors to the pathophysiology of OLP and the risk of transformation to OCC [13]. Specifically, IL-13, known to exacerbate tissue damage in chronic inflammatory diseases [16], has shown elevated expression levels in both OLP and OCC patients [17–20], suggesting its pivotal role in disease progression. Consequently, the measurement of interleukin concentrations in OLP could serve as a valuable tool for clinical assessment of patients [13]. However, the establishment of a definitive causal relationship between OLP and OCC, mediated by inflammatory cytokines, remains a challenge due to potential confounding factors and reverse causality inherent in conventional observational studies.

To overcome these challenges, Mendelian randomization (MR) presents a powerful tool. Utilizing genetic variations related to specific exposures as instrumental variables, MR can determine their causal effects on outcomes [21]. This approach leveraging genetic markers that are randomly distributed and established from conception, inherently has a lower risk of the usual confounding and reverse causality seen in observational studies [22]. As a result, over the past decade, MR has been increasingly used to provide more rigorous causal effect estimates for various risk factors of different health outcomes [23–26]. Furthermore, to explore mediating pathways, the multivariable MR (MVMR) method has been developed, offering significant reductions in biases compared to traditional multivariate methods [27]. This methodological innovation enables a more precise understanding of the pathways leading from OLP to OCC, potentially identifying novel preventive and therapeutic targets.

In this study, we utilized a four-step MR analysis founded on comprehensive statistical data from a large-scale genome-wide association study (GWAS), to evaluate the intermediary function and causal link of inflammatory cytokines in the risk between OLP, OL and OCC.

## Methods

### Overview of MR and its assumptions

**Figure 1:**
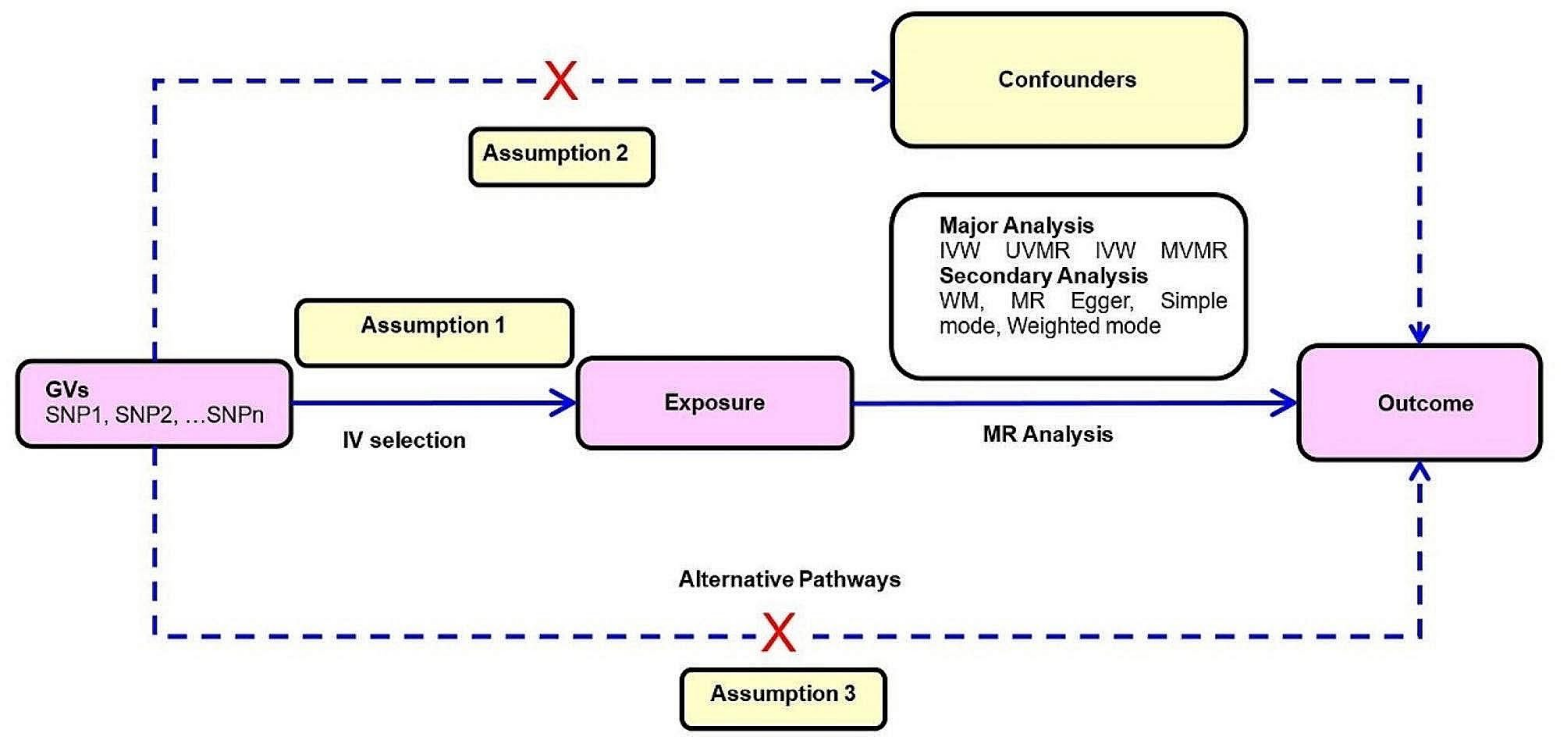
Overview of MR and its assumptions. **Notes**: There are three key assumptions for the MR study. Assumption 1: the genetic variants (GVs) selected as instrumental variables (IVs) should be robustly associated with the exposure; Assumption 2: the used IVs should not be associated with any potential confounder; Assumption 3: the GVs should influence the outcome risk merely through the exposures, not via any alternative pathway. **Abbreviations**: GVs = genetic variants; IV = instrumental variable; IVW = inverse variance weighted; MR = Mendelian randomization; MVMR = multivariable Mendelian randomization; SNP = single-nucleotide polymorphism; UVMR = univariable Mendelian randomization; WM = weighted median.

MR provides unconfounded estimates and overcomes the limitations of observational studies given three key assumptions are satisfied: (1) instrumental variables must be robustly associated with the exposure in question, (2) the instrumental variables must not be linked in any way to confounding variables in the relationship between genetic variants and the outcome of interest, and (3) the instrumental variables merely affect the outcome of interest via their association with the exposure of interest, with no alternative pathways coming into play (Fig. 1) [28].

### Overall study design

**Fig. 2:**
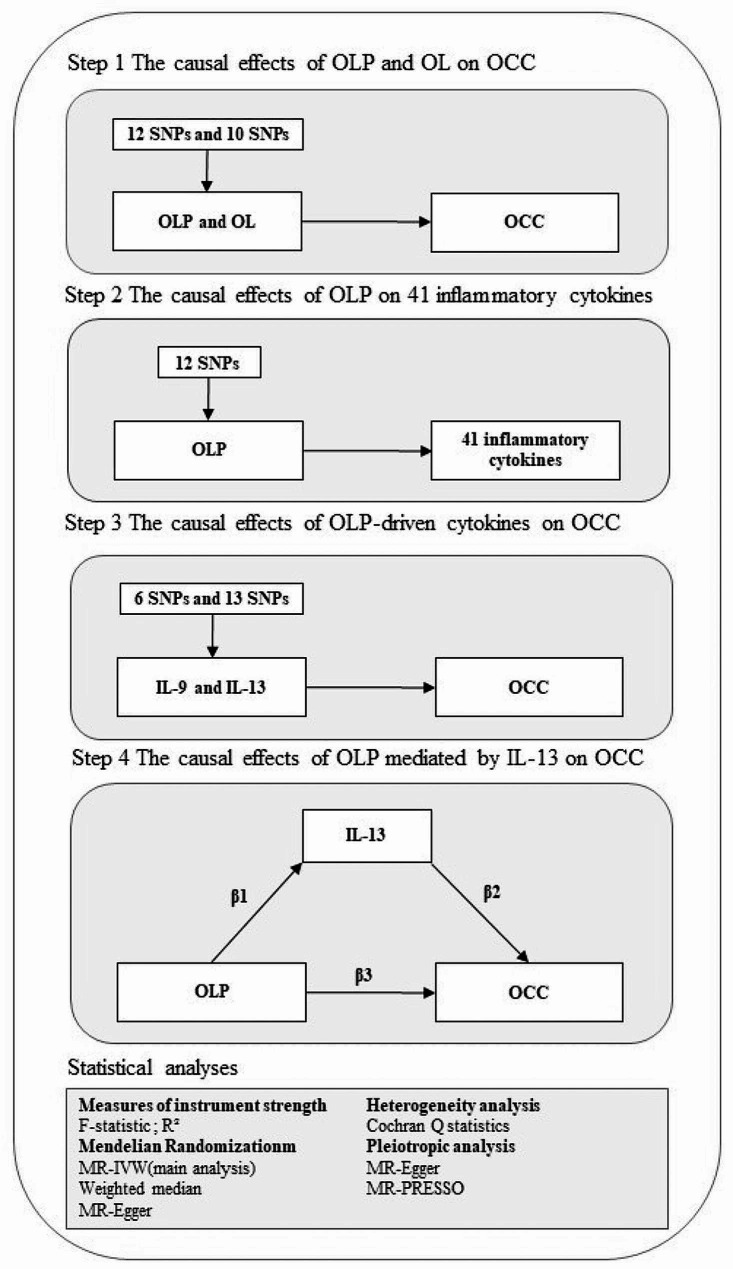
The overall framework of the proposed method **Notes**: The total effect of OLP on OCC, β3, was derived using univariable MR (i.e. genetically predicted OLP as exposure and OCC as outcome). The indirect effect using a two-step approach (where β1 is the total effect of OLP on IL-13, and β2 is the effect of IL-13 on OCC adjusting for exposure) and the product method (β1 × β2). Proportion mediated was the indirect effect divided by the total effect (β1 × β2/β3). **Abbreviations**: OLP = oral lichen planus; OL = oral leukoplakia; OCC = oral cavity cancer; IL-13 = interleukin-13; IL-9 = interleukin-9; IVW = inverse variance weighted; SNP = single-nucleotide polymorphism.

Figure 2 illustrates the methodological framework of this study, structured around a four-step MR analysis. In step 1, we assess the potential causal relationship between OLP, OL and the risk of OCC. This assessment utilizes OLP or OL GWAS data from the FinnGen r9 as the exposure and OCC GWAS data from MRC-IEU as the outcome variable, thus conducting a preliminary analysis (Sect. 2.3). Next, 12 SNPs linked to OLP and 10 SNPs linked to OL are identified as robust instrumental variables (Sect. 2.4). Employing the univariable MR (UVMR) analysis, primarily using the inverse variance weighted (IVW) approach, we estimate the direct effect of OLP or OL on the risk of OCC (Sect. 2.5). In step 2, the focus shifts to the causal impact of OLP on 41 inflammatory cytokines, mirroring step 1 but with the outcome variable replaced by GWAS data for these cytokines. Step 3 utilizes the UVMR analysis to examine the influence of OLP-driven cytokines on OCC risk, positing interleukin-13 (IL-13) as a potential mediator. In step 4, the MVMR analysis is employed to validate and quantify IL-13’s mediating effect on the OLP-OCC relationship (Sect. 2.6). To ensure the validity and robustness of our findings, heterogeneity, pleiotropy, and sensitivity analyses are conducted (Sect. 2.7).

### Data source

In this study, the OLP dataset was sourced from the Finnish R9 database, including a total of 377,277 individuals of European descent. This comprised 5,791 individuals diagnosed with OLP and 371,486 serving as control subjects. The OL dataset, on the other hand, comprised 2,382 individuals diagnosed with OL and 374,895 serving as control subjects. Additionally, data on 41 inflammatory cytokines were obtained from a GWAS study involving 8,293 Finnish individuals [29]. The outcome dataset, derived from the MRC-IEU database, consisted of 1,223 cases of OCC and 2,298 controls, all classified according to the International Classification of Diseases 10th Revision (ICD-10) criteria [30]. Our analysis targeted participants of European descent, including those diagnosed with OCC. Cancer cases were identified using the following ICD-10 codes: oral cavity cancer (C02.0–C02.9, C03.0–C03.9, C04.0–C04.9, and C05.0–C06.9). A more detailed description of the clinical variables included in the cohort is provided in Table S1.

### Genetic instruments

Genetic instruments closely associated with OLP were identified by setting a genome-wide significance threshold of *p* < 5 × 10^− 8^. To mitigate the effects of linkage disequilibrium, SNPs were clustered using PLINK version 1.90, with parameters clump-kb = 10,000 and clump-r2 = 0.001 [31]. Given the limited availability of SNPs for OL and the 41 inflammatory cytokines under consideration, a more relaxed threshold of *p* < 5 × 10^− 6^ was employed, along with the same parameters for adjusting linkage disequilibrium (clump-kb = 10,000 and clump-r2 = 0.001) [32]. Furthermore, each SNP’s R^2^ value was utilized to measure the variance in exposure, and the strength of these instruments was evaluated using the F-statistic, where SNPs with an F-value > 10 were considered effective instruments [33].

### UVMR Analysis

In line with the overall design steps described above, this study carried out UVMR analysis. To address potential horizontal pleiotropy, the study utilized three MR methods from the “TwoSampleMR” package for an integrated analysis: Inverse Variance Weighting (IVW), Weighted Median, and the MR-Egger method. The IVW method combines the Wald ratio of each SNP to deduce genetic causation, operating under the assumption that all SNPs are valid instrumental variables without horizontal pleiotropy [34]. Due to its advantages in providing high efficiency and precise estimates, the IVW method was chosen as the primary analysis method. On the other hand, the Weighted Median MR method aims to adjust the MR estimates of individual variables for accuracy, calculating the overall MR estimate as the median and using the bootstrap method to calculate standard errors [35]. The MR-Egger method performs regression analysis of the correlation between SNP-exposure and SNP-outcome, with each item weighted by the precision of the SNP-outcome correlation [36]. Given the different assumptions these methods make about horizontal pleiotropy, comparing their results can enhance the study’s reliability. Results were deemed statistically significant when the p-value was below 0.05. To further validate the findings, inflammatory cytokines that exhibited a causal association in both steps of the UVMR analysis were identified as potential mediators.

### MVMR analysis

In order to elucidate the mediation role of OLP-driven inflammatory cytokines in the influence of OLP on OCC, this research adopted the MVMR method for validation [37]. Initially, the direct effect of OLP on OCC in step 1 was determined, represented as β3. Subsequently, by estimating the direct impact of OLP on 41 inflammatory cytokines in step 2, the coefficient β1 was obtained. Afterward, considering the genetic effects of OLP, the impact of OLP-driven inflammatory cytokines on OCC risk was assessed using MVMR, yielding the coefficient β2. For quantifying OLP’s indirect influence on OCC via these inflammatory cytokines, the product-of-coefficients method was predominantly employed, wherein the multiplication of β1 and β2 represents the indirect impact of OLP on OCC through these cytokines. Finally, the proportion of the total effect mediated by inflammatory cytokines was assessed using the ratio of the indirect to the total effect (β1 × β2/β3) [38].

### Heterogeneity, Pleiotropy, and sensitivity analysis

Heterogeneity was quantified using the Cochran Q statistic. Specifically, when *P* > 0.05, suggesting no significant heterogeneity, analysis was conducted using a fixed-effect model. Conversely, if heterogeneity was significant (*P* < 0.05), a random-effects model was employed. In parallel, horizontal pleiotropy was assessed and addressed using MR-Egger and MR-PRESSO. If either of these methods showed *P* < 0.05, it was considered indicative of potential horizontal pleiotropy. To eliminate horizontal pleiotropy in the results, we used the “MR-PRESSO Outlier Test” to identify and exclude detected abnormal SNPs. Following this step, after excluding abnormal SNPs, MR analysis and horizontal pleiotropy testing were repeated until horizontal pleiotropy was completely removed (*P* > 0.05). Furthermore, additionally, a “leave-one-out” sensitivity analysis was conducted to ascertain the potential biasing effect of a single SNP on MR estimation.

## Results

### Eligible SNPs

In the MR analysis considering OLP and OL as an exposure, 12 SNPs and 10 SNPs with genome-wide significance were selected. Additionally, for OLP-driven inflammatory factors IL-9 and IL-13, respectively, 6 and 13 SNPs with genome-wide significance were identified. Table S2 provides detailed data on these aspects. The calculated F-statistics for the selected SNPs related to OLP, IL-9, and IL-13 all exceeded the conventional threshold of 10 (Table S2), indicating that these selected SNPs may be reliable representatives of OLP or IL-9 and IL-13.

### UVMR analysis of the causal effects of OL and OLP on OCC

The step 1 UVMR analysis results revealed a significant causal link between genetically forecasted OLP and increased risk of OCC. In particular, the main IVW method analysis indicated a harmful link between OLP and OCC (OR = 1.417, 95% CI = 1.167–1.721, *p* < 0.001), whereas no significant relationship was observed between OL and OCC (OR = 1.229, 95% CI = 0.832–1.814, *p* = 0.300). These findings were further validated by the Weighted Median and MR-Egger methods (Table 1).

**Table 1:** UVMR analysis of the causal effects of OL and OLP on OCC.

Outcome: OCC	OR	95%CI	*P*-value
Exposure: OL			
IVW	1.229	0.832–1.814	0.300
Weighted median	1.072	0.754–1.525	0.697
MR-Egger regression	0.749	0.303–1.850	0.548
Exposure: OLP			
IVW	1.417	1.167–1.721	0.001<
Weighted median	1.380	1.067–1.785	0.014
MR-Egger regression	1.158	0.780–1.719	0.484

Abbreviations: UVMR = univariable Mendelian randomization; IVW = inverse variance weighted; OCC = oral cavity cancer; OL = oral leukoplakia; OLP = oral lichen planus; CI = confidence interval; OR = odds ratio;

**Table 2:** MVMR analysis of the causal effects of OLP mediated by IL-13 on OCC.

Exposure	Inverse variance weighted		
	OR	95%CI	*P*-value
OLP	0.986	0.766–1.268	0.910
IL-13	1.437	1.139–1.815	0.002

Abbreviations: MVMR = multivariable mendelian randomization; OLP = oral lichen planus; IL-13 = interleukin-13; OCC = oral cavity cancer; CI = confidence interval; OR = odds ratio.

**Table 3:** Heterogeneity and pleiotropy analysis

Exposure	Outcome	nSNP	Heterogeneity			MR-Egger		MR-PRESSO
			Method	Cochran’s Q	*P*-value	Egger-intercept	*P*-value	*P*-value
OLP	OCC	12	IVW	10.522	0.484	0.045	0.277	0.515
OLP	IL-13	11	IVW	8.109	0.618	0.012	0.481	0.639
OLP	IL-9	11	IVW	9.748	0.463	0.022	0.196	0.450
IL-13	OCC	8	IVW	8.863	0.263	0.104	0.085	0.399

Abbreviations: IVW = inverse variance weighted; OLP = oral lichen planus; OCC = oral cavity cancer; IL-13 = interleukin-13; IL-9 = interleukin-9; SNP = single-nucleotide polymorphism;

### UVMR analysis of the causal effects of OLP on 41 inflammatory cytokines

**Figure 3:**
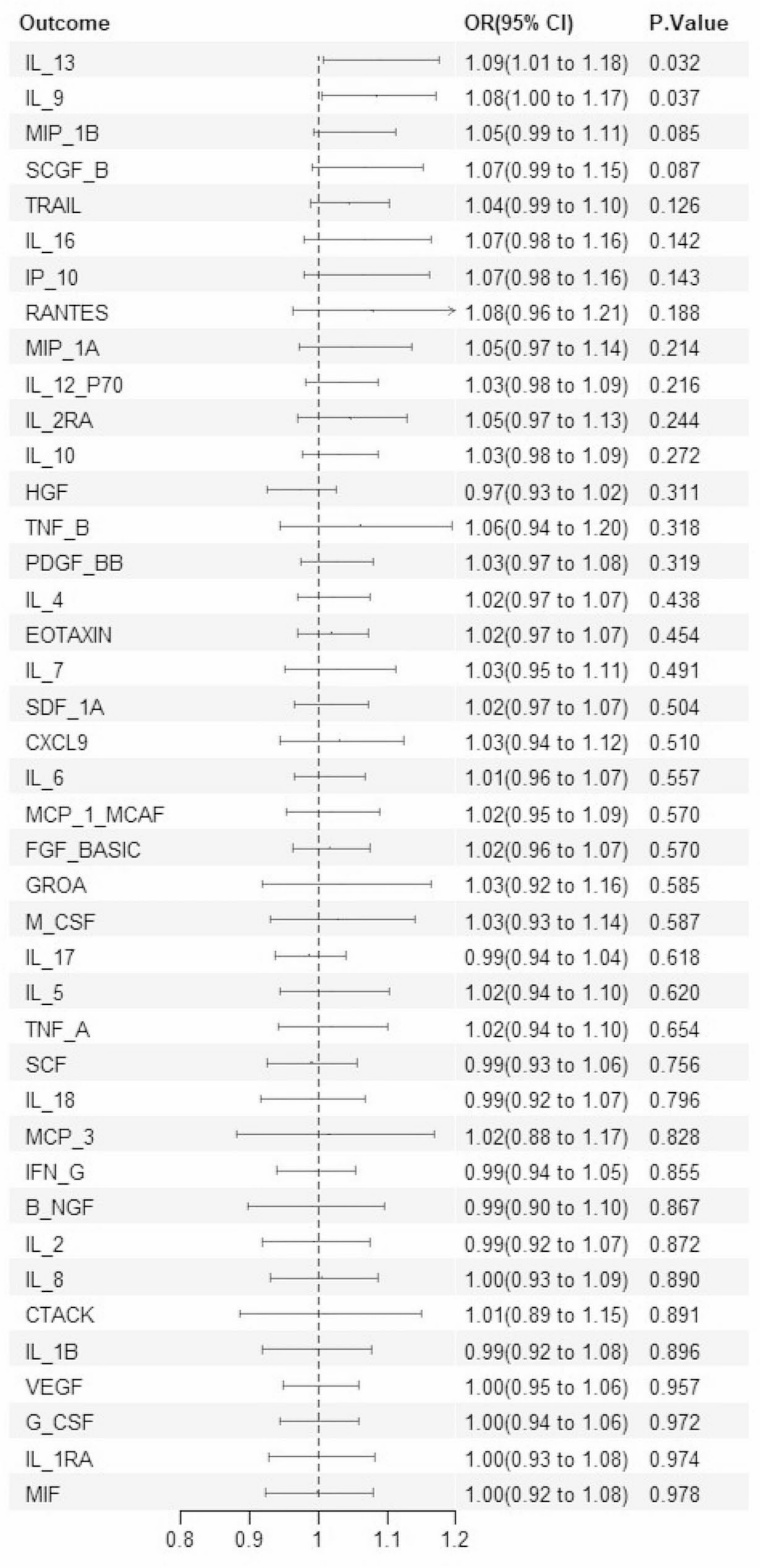
UVMR analysis of the causal effects of OLP on 41 inflammatory cytokines Notes: Association between genetically predicted OLP and 41 infammatory cytokines, using inverse variance weighted as the primary analytical method. OLP = oral lichen planus; CI = confidence interval; OR = odds ratio.

In step 2 UVMR analysis, two inflammatory cytokines, IL-13 and IL-9, were identified among the 41 cytokines as influenced by OLP. Specifically, genetic predictions using the IVW method revealed that elevated levels of IL-13 (OR = 1.088, 95% CI: 1.007–1.175, *P* = 0.032) and IL-9 (OR = 1.085, 95% CI: 1.005–1.171, *P* = 0.037) were significantly associated with OLP (Fig. 3), with these associations being largely confirmed by two other MR methods (Table S3).

### UVMR analysis of the causal effects of OLP-related inflammatory cytokines on OCC

**Figure 4:**
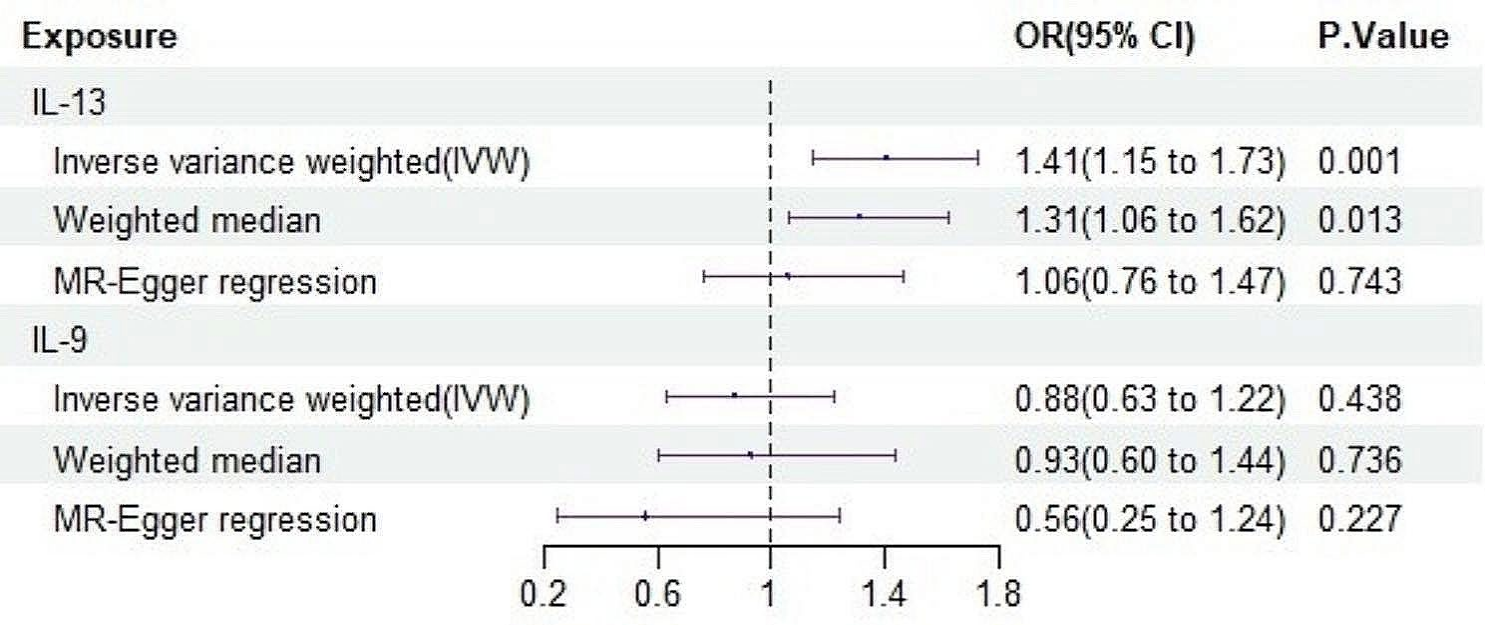
UVMR analysis of the causal effects of OLP-driven inflammatory cytokines on OCC Notes: Association between genetically predicted OLP-driven inflammatory cytokines and OCC, using inverse variance weighted as the primary analytical method. OLP = oral lichen planus; OCC = oral cavity cancer; CI = confidence interval; OR = odds ratio;IL-13 = interleukin-13; IL-9 = interleukin-9

In step 3, the UVMR analysis confirmed the causal link between OLP-driven inflammatory cytokines and the heightened risk of OCC. The findings indicated a significant causal connection between elevated IL-13 levels and an increased risk of OCC (OR = 1.408, 95% CI: 1.147–1.727, *P* = 0.001), as substantiated by results obtained from two additional MR methods (Fig. 4). Given the significant positive correlation of IL-13 with both OLP and OCC in steps two and three, it was identified as a potential mediating factor for further MVMR analysis.

### MVMR analysis of the causal effects of OLP mediated by IL-13 on OCC

In step 4 MVMR, IL-13 was established as a mediator between OLP and the increased risk of OCC. Our results demonstrate that, after adjusting for OLP, the correlation between genetically predicted IL-13 levels and a high risk of OCC remained strong (OR = 1.437, 95% CI = 1.139–1.815, *P* = 0.002) (Table 2), with a mediation effect of 8.13%. Furthermore, after adjusting for IL-13, the OR for the high risk of OCC associated with genetically predicted OLP was reduced from 1.2 to 0.986, rendering it insignificant (*P* > 0.05) (Table 2). These results indicate that IL-13 is the true mediating factor between OLP and a high risk of OCC.

### Heterogeneity, Pleiotropy, and sensitivity analysis

During the heterogeneity, pleiotropy, and sensitivity analysis, we observed that the heterogeneity of all results was not significant (*P* > 0.05) (Table 3). Furthermore, MR-Egger and MR-PRESSO tests did not detect any significant level of pleiotropy (*P* > 0.05) (Table 3). To further elucidate these positive results, we displayed the corresponding scatter plots in Figure S1 and provided forest plots in Figure S2. Using the “leave-one-out” sensitivity analysis method, we found that these causal relationships were essentially unaffected by any key SNPs (Figure S3). Lastly, the funnel plot in Figure S4 shows a fundamentally symmetrical distribution, indicating the absence of apparent publication bias.

## Discussion

To the best of our knowledge, this is the first study to explore the causal relationship between OLP and the risk of OCC mediated by IL-13 within an MR framework. The results revealed a significant causal association between OLP and an increased risk of OCC. Further analysis indicated a strong correlation between this increased risk and IL-13 levels, suggesting that IL-13 likely serves as a crucial mediator in the association between OLP and increased OCC risk. Specifically, IL-13 was responsible for mediating 8.13% of the OLP’s impact on OCC risk. These findings underscore the clinical importance of monitoring potential OCC risks in patients with OLP, which is crucial for the timely prevention and control of OCC.

In prior observational studies, extensive clinical follow-up research has demonstrated that patients with OLP exhibit a significantly increased risk of developing OSCC, a finding that corroborates the results of our MR study [39–43]. Nonetheless, factors such as smoking and Hepatitis C Virus infection have been identified as confounders that significantly elevate the risk of OLP progressing to OSCC, thereby compromising the reliability of clinical follow-up study outcomes to some extent [12, 41]. Despite these confounders, our MR analysis indicates a significant causal relationship between OLP and an elevated risk of OSCC, even after excluding these factors. This conclusion is supported by a 33-year cohort study, which suggests that the malignant transformation of OLP is not influenced by smoking [42]. Although tobacco use and alcohol consumption are major risk factors for OSCC, discussing these factors with OLP patients remains crucial in certain contexts. Furthermore, tissue samples from patients with OLP have shown elevated levels of IL-13 mRNA compared to control groups [17]. Similarly, an increase in IL-13 levels was observed in patients with OSCC [19]. These findings align with the outcomes of our MR analysis, bolstering the potential role of IL-13 in the transition from OLP to OSCC. However, while traditional observational studies have uncovered associations between these variables, they fall short in robustly investigating the causal relationships.

This research utilized genetic forecasting techniques and found that IL-13 facilitates a direct causal link between OLP and the heightened risk of OCC, contributing to 8.13% of this mediation, aligning with other studies and less likely to be influenced by confounding biases or reverse causal effects. Chronic inflammation, such as OLP, plays a key role in the development of cancer. The balance between pro-inflammatory activity and anti-tumor immunity within the tumor microenvironment is crucial as it determines the proliferation or apoptosis of cancer cells [44]. Inflammatory states are impacted by cytokines from nearby immune cells, tumor cells, and other tumor-related host cells, and these inflammatory cytokines are key in tumor progression, neovascularization, immune monitoring, and metastasis [45, 46]. IL-13, a critical type 2 inflammatory cytokine originating from T cells, is engaged in the aforementioned processes [47]. By binding to its receptors IL-13Ra1 and IL-13Ra2, it activates downstream signaling cascades, influencing the proliferation, inhibition, or apoptosis of certain tumor cells [48]. Existing research suggests that IL-13 and its receptors are linked to the advancement of multiple cancer types [49, 50]. Particularly in patients with OSCC, the expression levels of IL-13 and IL-13Ra2 are elevated [18–20]. However, these findings were largely based on observational studies. Utilizing the MR analysis, this research further uncovered the mediating function of IL-13 in the heightened risk of OCC due to OLP. IL-13, as a critical mediator in inflammatory responses, intensifies tissue damage during chronic inflammatory diseases and engages in widespread regulation of gene expression [16]. Previous studies have shown that the mRNA levels of IL-13 are elevated in OLP patients, potentially increasing the risk of damage and fibrosis to oral mucosal cells [17]. Recent studies discovered that IL-13 receptor-targeted cytotoxin exhibits significant cytotoxicity in OSCC cell lines, but is non-toxic to normal oral fibroblasts [18]. Despite this, most research in the OCC field focuses on the levels of inflammatory cytokines in the plasma, with the relationship between IL-13 and OLP as well as OCC has not been adequately reported [51–53].

The significance of IL-13 in tumors may be closely associated with the actions of IL-4 and its receptors. IL-4, a multifunctional peptide structurally similar to IL-13, influences various cell types, notably playing a significant role in the proliferation, survival, and metastasis of epithelial tumor cells [54]. These cytokines primarily signal through the type II IL-4R, composed of IL-4Rα and IL-13Rα1, which is expressed in a wide array of epithelial tumor cells [55, 56]. Based on the type II IL-4R, IL-13 predominantly activates the Stat6 pathway upon binding with IL-13Ra1, a pathway also activated by IL-4 upon binding to the same receptor [57]. Research indicates that in mice with IL-4Ra gene deficiency, the transmission of IL-13 signals relies on IL-13Ra2, potentially leading to the development of precancerous lesions in colon cancer [58]. Conversely, in the absence of IL-13Ra2, IL-13 may enhance signal transduction through the type II IL-4R, thereby activating the IL-13-induced apoptotic pathway [46]. Furthermore, the high expression of IL-4Ra and IL-13Ra2 in various tumor cells presents the potential for selectively delivering toxins or lytic peptides to reduce tumor burden [59]. In summary, IL-4 and IL-13 and their receptors play a significant role in tumor cell biology. While the signaling pathways activated by these Th2 cytokines are complex and require further elucidation, they offer potential new targeted approaches for cancer-specific behaviors.

There are still limitations in this study: First, the research’s reliance on GWAS samples from individuals of European ancestry limited its generalizability to non-European groups. Secondly, the absence of GWAS data for OLP patients adhering to diverse diagnostic standards hindered the study’s capacity for detailed subgroup analysis of the malignant transformation risks in various OLP subtypes. Thirdly, the analysis method primarily utilized compiled statistical data rather than raw data at the individual level, diminishing its capacity to distinguish between early and late stages of OCC or examine other nonlinear relationships.

## Conclusion

In this study, a causal link between OLP and increased OCC risk was established through MR analysis of GWAS data. This analysis revealed that the association might be facilitated through the mediation of IL-13. The discovery suggested that IL-13 could be a potential target to diminish the malignant progression from OLP to OCC. Yet, the detailed mechanism by which inflammatory cytokines augment OCC risk by modulating OLP remains insufficiently explained. This lack of knowledge underscored the necessity for interventional studies to evaluate the effectiveness of treatments targeting inflammatory cytokines in lowering the risk of OLP patients progressing to OCC.

### Electronic supplementary material

Below is the link to the electronic supplementary material.

Supplementary Material 1

Supplementary Material 2

## Data Availability

In this study, the aggregated data for exposures and outcome were predominantly sourced from the FinnGen r9, IEU OpenGWAS project (https://gwas.mrcieu.ac.uk/) and associated publications, including OLP, 41 inflammatory cytokines[30] and Oral and Oropharyngeal Cancer[31]. Other datasets generated and/or analyzed during the current study are publicly accessible and have been included in the published article and its supplementary information files.
