# Supplementary material for: Inflammatory cytokines mediating the effect of oral lichen planus on oral cavity cancer risk: a univariable and multivariable mendelian randomization study
Source: BMC Oral Health. 2024 Mar 22;24:375. doi: 10.1186/s12903-024-04104-0 (PMC10958829; doi:10.1186/s12903-024-04104-0)
Supplement: Supplementary file 2 — Supplementary Material 2 [file 12903_2024_4104_MOESM2_ESM.docx]

## **Supplement figure 1. MR scatter plot**

| 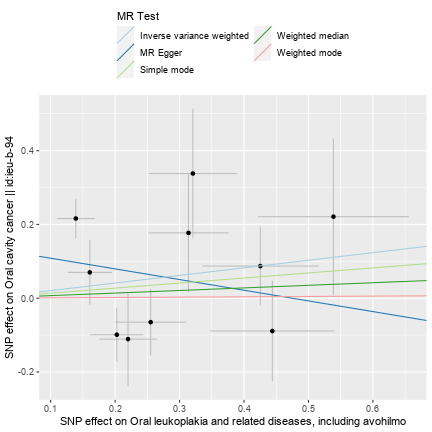 | 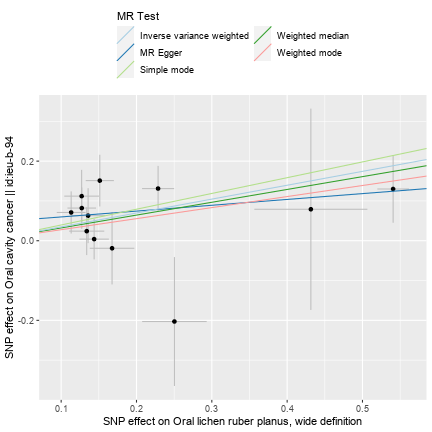 |
| --- | --- |
| 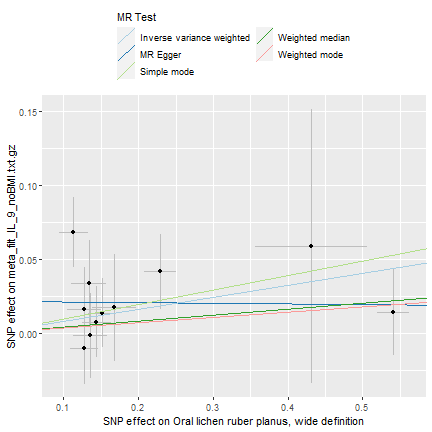 | 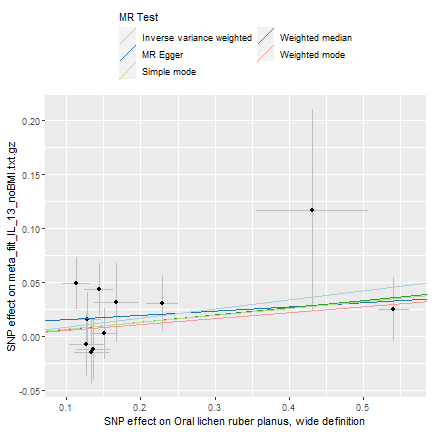 |
| 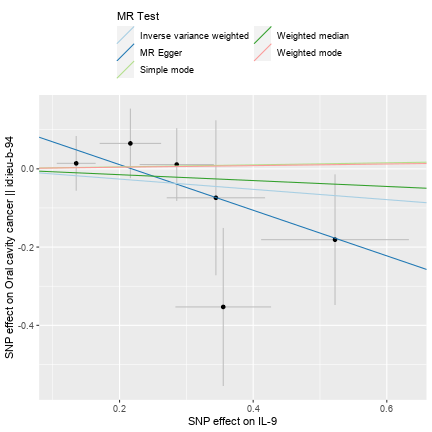 | 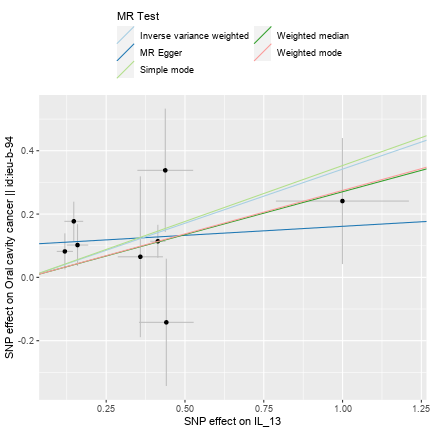 |

## **Supplement figure 2. MR forest plot**

| 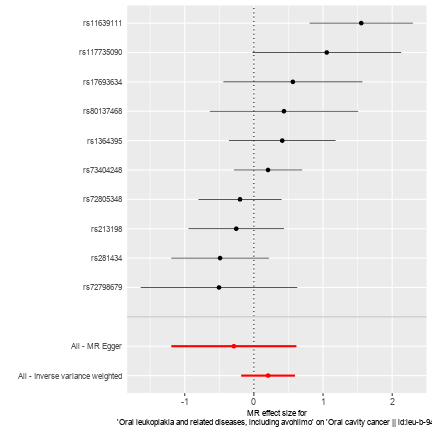 | 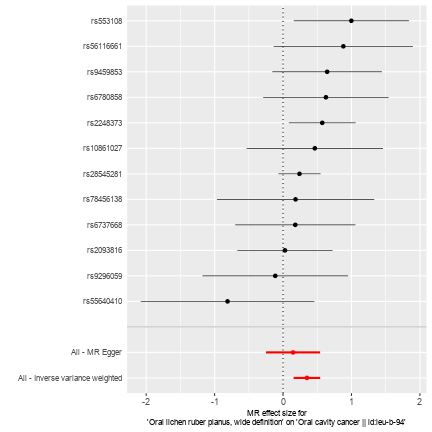 |
| --- | --- |
| 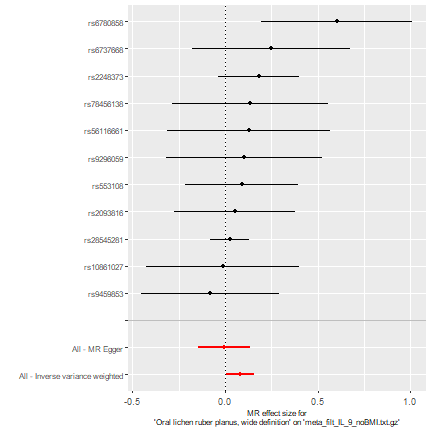 | 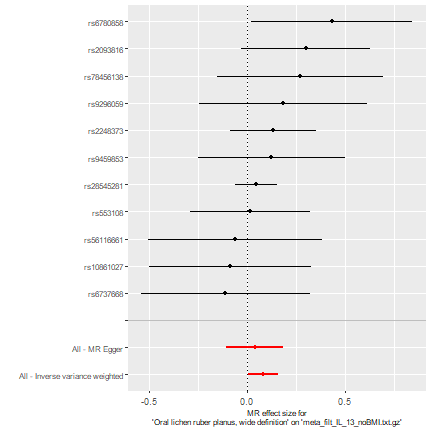 |
| 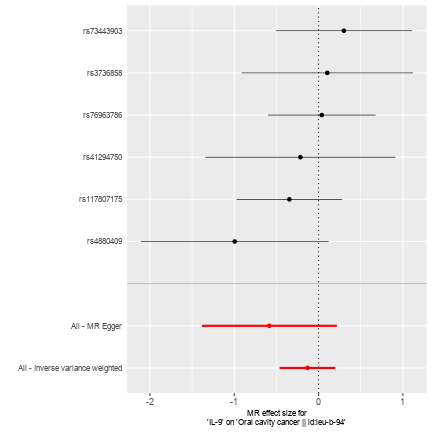 | 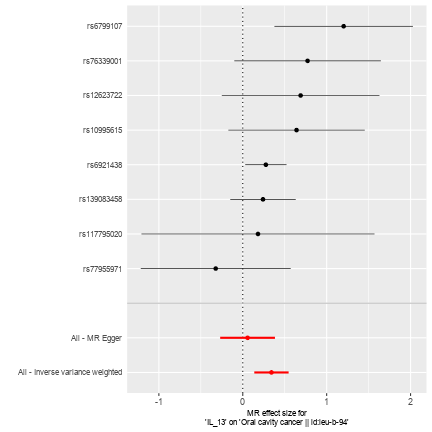 |

## **Supplement figure 3 MR_leaveOneOut_plot**

| 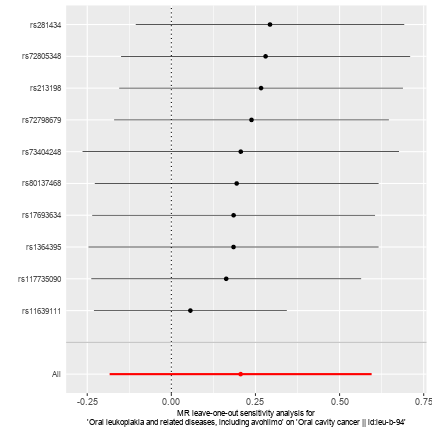 | 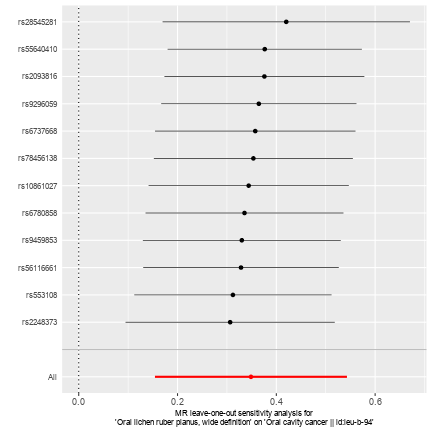 |
| --- | --- |
| 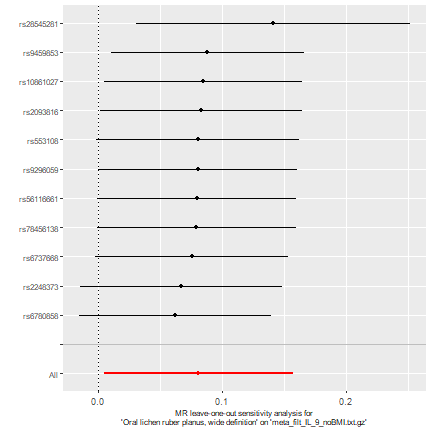 | 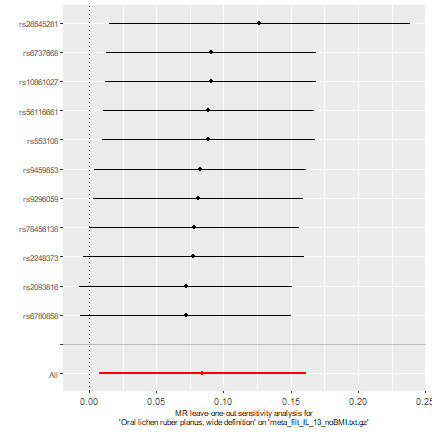 |
| 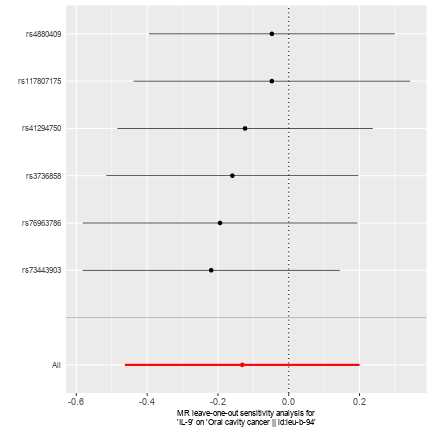 | 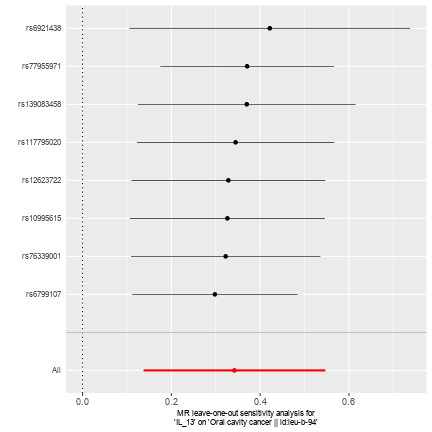 |

## **Supplement figure 4 MR_funnel_plot**

| 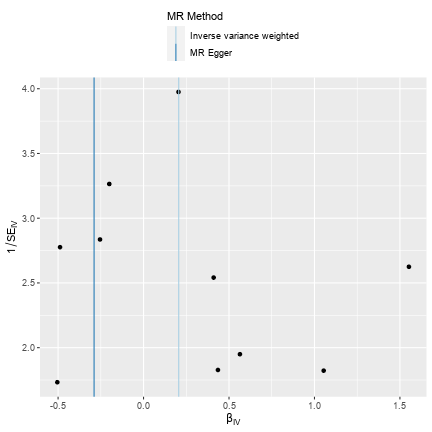 | 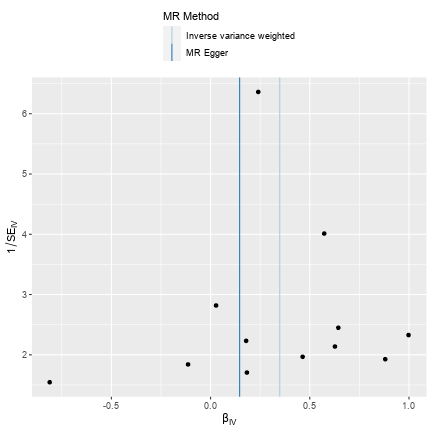 |
| --- | --- |
| 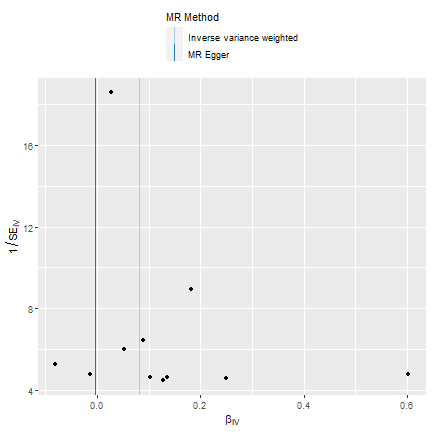 | 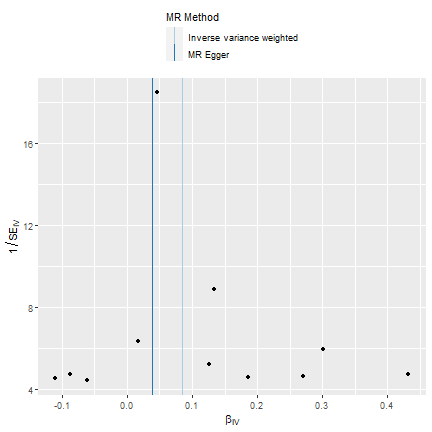 |
| 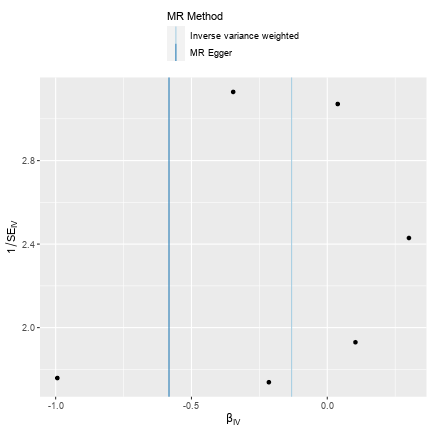 | 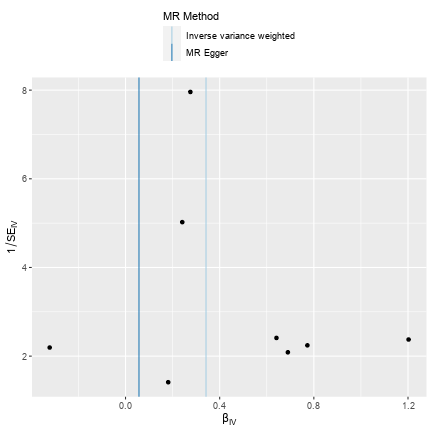 |
